# Beyond the Wall: Death Education at Middle School as Suicide Prevention

**DOI:** 10.3390/ijerph17072398

**Published:** 2020-04-01

**Authors:** Ines Testoni, Elisa Tronca, Gianmarco Biancalani, Lucia Ronconi, Giovanna Calapai

**Affiliations:** 1 Department of Philosophy, Sociology, Education and Applied Psychology (FISPPA), University of Padova, Via Venezia 14, 35131 Padua, Italy; tronca.elisa@gmail.com (E.T.); gianmarco.biancalani@unipd.it (G.B.); l.ronconi@unipd.it (L.R.); 2 Alma.Thi Association, 36016 Thiene, Italy; giovanna.calapai@gmail.com

**Keywords:** adolescence suicide prevention, death education, psychodrama, photovoice

## Abstract

This study investigates the psychological effects of participation in Death Education (DeEd) by middle school children in two towns in northeast Italy in which suicides occur to a greater extent than in the rest of the region. The aims of the project “Beyond the Wall” were inherent to the prevention of suicide, address existential issues and enhance the meaning of life through positive intentions for the future and reflection on mortality. It involved eight classes (150 students in four classes in the experimental group; 81 in four classes in the control group) engaging with films, workgroup activities, photovoice and psychodrama. The constructs of resilience, emotional competency and psychological well-being were monitored with the Resilience Scale for Adolescents, the Hopelessness Scale for Children, the Alexithymia Questionnaire for Children and the Stirling Children’s Well-being Scale. The DeEd intervention was found to be significantly related to some of the variables investigated, improving the students’ ability to recognise emotions and communicate them verbally while maintaining stable initial characteristics, such as psychological well-being and positive expectations for the future.

## 1. Introduction

In the US, suicide is the second leading cause of death among pubescent children and adolescents between the ages of 10 and 19 [1]. In Italy, suicide is the third most frequent cause of death between the ages of 15 and 24, after road accidents and cancer. The methods most frequently used by both boys and girls from 2000 to 2002 were hanging (48% boys; 26% girls), firearms (20% boys; 14% girls) and jumping from a high place (18% boys; 49% girls). Significant differences were also observed between the three Italian macro-regions: 2.63 ± 0.60 in the north, 2.07 ± 0.35 in the centre and 2.19 ± 0.40 in the south. This can be explained by the protective role of the networks of family and friends that are strong in southern Italy. Boys die of suicide more frequently than do girls, at a ratio of 2.1:1 [2]. It is difficult to establish a cause for these traumatic events; however, in the 15–19 age group, there is a higher frequency of suicides in schools, often going together with problems such as self-esteem and relationships with friends and family (33%), depression (13.8%) and family problems (5.6%) [3].

Because of this, many primary prevention initiatives have been implemented in Europe with the help of educational agencies worldwide. For example, school-based suicide prevention programmes promoted by the American Foundation for Suicide Prevention, the Life Skills Education Programme [4] and the European Regions Enforcing Actions Against Suicide (EUREGENAS) programme [5] have tried to transform schools into a formative space to improve protective factors that reduce students’ frustration levels and the conditions that cause social isolation [6,7,8,9,10,11]. However, this may be insufficient due to the lack of existential reflection on the relationship between death-related thoughts and the meaning of life [12]. In fact, in Western society, the postmodern era has produced exponential growth in well-being, together with a progressive secularisation of culture and the total medicalisation of death [13]. The systematic removal from social life of death and dying, which are increasingly relegated to healthcare facilities, is accompanied by the rarefaction of existential reflections and educational paths centred on the value of life and the sense of finitude [14]. Despite the fact that many authors have underscored the importance of raising public awareness of existential issues during school activities [15,16], the almost total absence of any form of rational discussion on mortality may abandon children, pubescent boys and girls and adolescents to irrational adherence to dangerous attitudes towards death [17]. All of this may contribute to a reduction in resilience towards facing such difficulties [18,19].

Death Education (DeEd) encompasses individuals’ needs to learn about death during their lifetimes, offering a conceptual basis to appreciate that a lifespan is limited and an opportunity to acquire a mature awareness of mortality and to understand that life must be preserved and protected [20]. Many studies confirm that consciousness of one’s own limits implies a greater valorisation of life and a better existential formation [21,22,23]. Indeed, many experiences show how students need to be able to express themselves freely and creatively about the meaning of life and death [24]. Unfortunately, adults, educators and parents have proven unwilling to answer children’s and adolescents’ questions on these issues, believing that they are protecting them from negative thoughts. Not used to reflecting on such a theme, they in turn transmit this inability to understand and process the subject to their children, thus increasing the horrific representations of death, or the view that there is a constant threat from killers, ghosts and monsters [25,26,27]. Consequently, pubescent youth and adolescents are left alone in their search for information about death and subject to the influence of the media, which offers unrealistic messages that do not help them manage this topic in a mature way [28,29,30,31]. All this can be particularly dangerous, especially when children believe that suicide can be a solution to their problems and cannot find a relational space in which to discuss their negative thoughts [32,33].

In contrast to this common perspective, DeEd can be taught in the context of formal schooling and approached from a perspective of suicide prevention or the elaboration of traumatic loss because it allows teachers to intervene in the representations of death and address the need to rebuild a sense of life. As some research has already shown, DeEd may be very useful in the management of suicide risk [34,35,36,37]; specifically, when students live in a space in which the trauma of suicide is present, it may transform the risk of the Werther effect into a positive elaboration of death-related thoughts aimed at valorising and reinforcing the meaning of life [38,39]. Indeed, it is important to consider the effect of proximity to suicide experiences and the implicit social construction of its acceptability that needs to be avoided in order to stop the imitative effects that result from identification and familiarity [40]. A classic example is the case of the Wittgenstein family, where three out of four brothers of the famous philosopher Ludwig died by suicide. The prevention interventions are exactly aimed to elaborate the traumatic experience of pursuing suicide, to avoid its following imitative effect.

The aim of DeEd is to provide information on death that is supported by the appropriate language to aid emotional understandings, trigger reflections on the meaning of life, strengthen critical thinking and support the sharing of experiential learning with friends and classmates [41]. A programme of this nature may be effective for ensuring teenagers’ good mental health because it allows them to consider death realistically, reducing both anguish and egocentric fantasies. Since it activates reflection on the sense of existence by exploring issues such as concerns about death and beliefs in the afterlife [18,42], DeEd makes it possible to manage the themes derived from suicide ideation, promoting discussion and raising awareness of the reality of death while reflecting on spirituality and transcendence [43].

The creation of educative spaces in which students can strengthen their ability to distinguish between different feelings may help support them in admitting that life is precious even if it is difficult, and that hurting or killing oneself does not solve problems [14,44,45]. From this perspective, the use of films, photography and artistic techniques, such as psychodrama, is useful for improving the development of not only the individual, by promoting positive changes, but also the entire community in which children live [46,47,48,49,50]. In particular, photovoice incorporates a series of group actions to promote a mobilisation by young people for community change [51,52,53]. From this perspective, in fact, as other experiences have shown, it is necessary to involve families and the community to support these educative activities [17].

This article presents the results of a study that was made possible by a DeEd project entitled “Beyond the Wall” (BW), which is similar to a previous project conducted under the title “Before I die I want to” [54]. The fundamental idea unifying both courses was to allow students to reflect on their own futures, their desires and the objectives they wanted to achieve before their deaths. BW involved four middle schools in a territory within the Veneto Region of northeast Italy, where suicide rates were higher than other regions and where the problem of juvenile suicide was particularly felt by all the communities. In fact, BW was promoted by the municipal administrations, which requested a specific intervention to prevent suicide.

## 2. Rationale for Research

The research was carried out using a quantitative methodology, specifically the use of pre- and post-intervention questionnaires.

## 3. Aims

The educational objectives of BW were to help young people to cope with being told of the suicide of students at their schools and to become aware of their negative emotions and their representations of death, to improve their ability to cope with such negative thoughts. Further requirements were that none of the activities addressing the issue of death would increase anxiety, fear or negativity, thus jeopardising the students’ resilience, well-being and positive expectations for the future. The fundamental hypothesis was that, through the intervention of DeEd BW, emotional skills could be developed, maintaining the psychological well-being of the participants and strengthening their future aspirations, despite the reflection on death. The study hypotheses were the following: (a) DeEd increases the ability to manage and process emotions, especially negative ones; (b) the DeEd reflections on the suicide choices of other students maintain a stable level of well-being among the students; and (c) DeEd increases resilience.

This research received approval from the Padova University Ethics Committee for Experimentation (No. 99C54686990FC9D4035B86F8FD999885) and followed the APA Ethical Principles of Psychologists and Code of Conduct and the principles of the Declaration of Helsinki. As required by these standards, all the aims and instruments were explained in a preliminary meeting to the parents of the participants, and their signed informed consent was obtained.

## 4. Participants and Activities

Participants were 150 students from eight classes in two middle schools (four classes in the experimental group and four classes in the control group). The roles of the classes had been determined according to the availability and interests of the teaching staff and the school administrations. During the first administration of the questionnaire, there were 82 participants in the experimental group and 70 participants in the control group; the latter did not participate in the course. During the second administration of the questionnaire, two students (one per group) were absent. Ultimately, the experimental group consisted of 81 students (38 girls and 43 boys) and the control group of 69 students (35 girls and 34 boys) aged between 12 and 14 years (M = 12.20, SD = 0.42). The two groups were comparable with regard to gender (χ^2^ = 0.22, df = 1, *p* = 0.642) and age (M = 12.23, SD = 0.43 in the experimental group and M = 12.16, SD = 0.41 in the control group; t = 1.10, df = 148, *p* = 0.274). The activities were divided into four meetings of two hours each, led by psychologists who had previously worked in death education. During these meetings, film viewing, psychodrama and photovoice activities were conducted, centred on positive expectations for the future and the consciousness of finitude. The Disney Pixar animated film *Coco*, set in Mexico during Día de Muertos, describes the adventures of young Miguel, who, to follow his passion for music, faces several obstacles. After viewing the film, psychodrama activities aimed at dramatising the role of *Coco* allowed participants to identify with the story, thereby internalising the message. The participants then engaged in photovoice activities. They were asked to take pictures regarding the theme “In my life I would like to”, to give then the opportunity to think about their dreams and future projects. Finally, workgroups were formed to create posters illustrating their emotions, their futures and their sense of life. All participants explained their photos, creating a legend for each picture with other members of the group, which were then presented to the whole class. 

## 5. Measures

The questionnaire was completed online by the students, in both the experimental and control groups, both before and after the intervention. Each student received an identification code to access the online questionnaire using credentials provided by the University of Padua in order to maintain anonymity. The following instruments were used:

The Resilience Scale for Adolescents (READ) [55] investigates the ability of adolescents to cope positively with traumatic events or difficult situations [56]. The original version consists of 28 items measured with a 5-point Likert scale. The Italian adaptation was developed through back translation of the Mexican version [57] and was composed of 22 items on a 5-point Likert scale (from 1 = not at all agreeable to 5 = very agreeable). The READ comprises five factors [55]. Family cohesion (FC), relates to shared values, family support and a positive future perspective (e.g., “In the family we share the opinion of what is important in life”) and has good reliability (pre-test α = 0.85, post-test α = 0.86). Social competence (SC) relates to the extroversion, social skills, good humour and the ability to start conversations and flexibility in social environments (e.g., “I easily make new friends”) and has adequate reliability (pre-test α = 0.76, post-test α = 0.74). Personal competence (PC) relates to self-esteem, self-efficacy, self-acceptance, hope, determination, life orientation and the ability to plan and organise daily routines (e.g., “When I have to choose between different options, I almost always know which is best for me”) and has adequate reliability (pre-test α = 0.66, post-test α = 0.73). Social resources (SR) relates to the perception and availability of external support, such as having many friends (e.g., “I have friends and family who care about me”) and has adequate reliability (pre-test α = 0.74, post-test α = 0.79). Finally, goal orientation (GO) relates to the individual’s preferences for planning and structuring his or her daily routine (e.g., “I know how to achieve my goals”) and has adequate to low reliability (pre-test α = 0.64, post-test α = 0.65).

The Hopelessness Scale for Children (HSC) [58] is composed of 17 items equipped with true/false response options, (e.g., “I can imagine what my life will be like in 10 years”) and has adequate reliability (pre-test α = 0.70, post-test α = 0.68). High scores (maximum = 17) reflect greater despair or negative expectations for the future, the latter of which is one of the three elements of cognitive distortion belonging to Beck’s model of depression [59]. High scores on the HSC appear to be predictive of suicide attempts [60].

The Alexithymia Questionnaire for Children (AQC) [61] measures the construct described by Sifneos [62] as the difficulty in identifying and communicating feelings, difficulty differentiating the somatic sensations experienced, a decrease in fantasy and imagination and an externally oriented cognitive style [63]. The questionnaire consists of 20 items and is based on a 3-point Likert scale (0 = not true; 1 = sometimes true; 2 = often true); in this study, the version of the scale validated in Italian was used. The original version is structured on three factors: difficulty in identifying feelings (DIF) (e.g., “When I feel bad, I do not know if I am sad, scared or angry”), which has adequate reliability (pre-test α = 0.69, post-test α = 0.77); difficulty in describing one’s feelings (DDF) (e.g., “It’s hard to explain how I feel”), which has low reliability (pre-test α = 0.60, post-test α = 0.69); and externally oriented thinking (EOT) (e.g., “It is important to understand how you feel inside”), which has very low reliability (pre-test α = 0.38, post-test α = 0.31).

The Stirling Children’s Well-being Scale (SCWBS) [64] measures psychological well-being and emotional development in children and evaluates the effectiveness of interventions and projects to promote their well-being in the last two weeks. The SCWBS is composed of 15 items and is structured on a 5-point Likert scale, with a minimum score of 12 and a maximum of 60 (α = 0.85). The questionnaire is divided as follows [65]: a positive emotional state corresponds to subjective hedonic well-being and is therefore related to feelings of optimism, joy and relaxation, as well as to satisfaction in interpersonal relationships and positive functioning (e.g., “I feel in a good mood”); a positive outlook corresponds to eudemonic well-being; that is, energy, clear thinking, self-acceptance, personal development, competence and autonomy (e.g., “I think I can be proud of many things”); and a social desirability indicator (e.g., “I have always told the truth”). Although this scale has three subcomponents, we decided to consider only the total score in the analysis, which showed good reliability (pre-test α = 0.89, post-test α = 0.89).

In this study, the version of the AQC scale validated in Italian was used [66], while for the other scales, as there was no Italian validation, back translations were made.

## 6. Data Analysis

To examine our overall expectations, i.e., that death education would generate improved scores in the experimental group compared to the control group, analysis of variance (ANOVA) was performed on each measure with a within-subjects factor of *time* (pre-test and post-test) and a between-subjects factor of group (experimental and control). At the pre-test stage, the two groups were compared on all study variables using t-tests and Pearson correlations between all measures. In examining the correlations, we classified effect sizes as small (0.10 to 0.25), medium (0.25 to 0.40) or large (over 0.40) [67]. Moderation analysis was carried out to evaluate the impact of DeEd intervention on the relationship between global alexithymia change (add the difference from pre-test to post-test for each factor) on the one hand and student’s well-being and resilience at the post-test on the other. All analyses were conducted using IBM SPSS Statistics Version 25 (IBM, Armonk, NY, USA) and the macro PROCESS for SPSS created by Preacher and Hayes for moderation analysis [68].

## 7. Results

At the pre-test stage, the two groups, experimental and control, showed a significant difference on two factors of the questionnaire that measure alexithymia: DDF (t = 2.53, df = 148, *p* = 0.013) and EOT (t = 2.45, df = 148, *p* = 0.016). Specifically, the experimental group started with higher levels than the control group on alexithymia, i.e., the inability to understand one’s emotions (Table 1). Moreover, during the pre-test, the two groups had significantly different scores on two components of resilience—SR (t = −2.62, df = 148, *p* = 0.010) and FC (t = −1.99, df = 148, *p* = 0.048)—and differences that were close to statistical significance on another component of resilience, SC (t = −1.91, df = 148, *p* = 0.058). Consequently, for resilience, the experimental group started at lower levels than the control group (Table 1).

Correlations between measures are reported in Table 2. We can observe positive correlations between alexithymia and hopelessness and between resilience and children’s well-being. On the contrary, there are also negative correlations between these two clusters of measures. In general, the values indicate a small-to-medium effect size of the relationship, except for children’s well-being, which shows a large effect size with hopelessness and with resilience.

The ANOVA results (Table 1) show a significant “time x group” interaction only for only two factors of alexithymia: (DDF) and (EOT). In particular, we observed a significant decline from pre-test to post-test in the experimental group (t = 2.39, *p* = 0.018 for DDF; t = 2.38, *p* = 0.019 for EOT) and no significant variation in the control group (t = −0.90, *p* = 0.368 for DDF; t = −0.76, *p* = 0.446 for EOT). Moreover, there was a significant main effect of time for one factor of alexithymia (DIF) and for two dimensions of resilience (SR and SC). On these measures, the students, in both the experimental and control groups, received lower scores after the intervention than before. Finally, there was a significant main group effect for one dimension of resilience, SR, for which the experimental group continued to receive lower scores than the control group in the post-test. There were no significant effects for the HSC or SCWB.

Finally, moderation analysis was performed to estimate the impact of alexithymia reduction on student’s well-being and resilience at the post-test. In each model, one model for the total score of well-being at the post test and five models for the five subscales of resilience at the post-test, we included the same measure at the pre-test as a covariate, the global alexithymia change as focal predictor and the death education intervention (coded as dummy variable: 1 = Yes and 0 = No) as moderator. Results show a significant moderation effect of death education, i.e., interaction of global alexithymia change by death education intervention, on well-being and on the subscale of resilience personal competence (Table 3). The conditional effects of global alexithymia change due to death education intervention show that only in the experimental group does an alexithymia reduction increase well-being and personal competence at the post test (B = 3.36, SE = 0.94, t = 3.57, *p* < 0.001 and B = 0.26, SE = 0.08, t = 3.25, *p* = 0.001, respectively), but not in the control group (B = −0.036, SE = 1.01, t = −0.35, *p* = 0.724 and B = −0.02, SE = 0.09, t = −0.20, *p* = 0.846, respectively).

## 8. Discussion

The BW project was conducted in two towns in which suicide rates are higher than in other areas of the same region of northeast Italy and involved young students from local schools. The project confirmed that it is possible to organise a death education intervention without worsening students’ well-being, positive expectations of the future or resilience. Notably, the study showed a change in alexithymia following the intervention. As with adults, alexithymia for pubescent youth is accompanied by hopelessness—a lack of hope for the future—and a decline in resilience and well-being. It is important to underscore that, in the pre-test, the experimental group had higher scores than the control group in terms of the difficulty they had in describing their feelings and externally oriented thinking. The intervention was actually conducted in the classes in which there was greater need, according to the evaluations of teachers and administrators, and there was thus no randomisation in the sample. Furthermore, the two factors that were significant over time for the group variables were difficulty in describing one’s feelings and externally oriented thinking, which were negatively correlated with the components of resilience and well-being. A significant decrease in these two components, as has occurred in the experimental group, also might have an important positive impact on resilience and psychological well-being. In this study, the pre-test correlations were included as preliminary analyses to confirm the relationships that had been shown in other studies between the constructs being examined. It was confirmed that difficulty in identifying one’s own emotions means less personal competence and more difficulty in directing one’s own objectives. Thus, difficulty in describing one’s feelings is linked to a decrease in resilience in all its aspects—that is, in the inability to deal positively with difficult situations in life. An outwardly oriented thought process decreases resilience, and a greater misunderstanding of one’s own emotions therefore seems to worsen psychological well-being. It is then noteworthy that, in the post-test, the experimental group showed a significant decrease in difficulty describing one’s feelings and externally oriented thinking, while in the control group, these variables remained unchanged. Consistent with the literature [38,45], the DeEd participants improved their ability to describe their feelings, decreased their externally oriented thinking, strengthened their attention toward internal dimensions and demonstrated their ability to speak about all of this with less difficulty. Although no significant interactions emerged in the resilience components, there was a decrease only in the three components of family cohesion, social competence and social resources in the control group, all of which instead remained constant in the experimental group. The intervention therefore did not lead to a worsening of resilience, i.e., the ability to deal positively with difficult events in one’s life. Furthermore, the construct of alexithymia correlated positively with hopelessness, and a decrease in alexithymia therefore leads to a lower risk of neuroticism and depression and to a positive impact on the construct of resilience [64,66]. In light of this, the intervention had positive effects by achieving the objectives, as supported by the literature [38,45].

On the basis of this it is possible to say that the death education intervention can be utilized to counteract the normalisation and familiarisation with suicide that censorship of suicide experiences can facilitate. In fact, the Beyond the Wall project has allowed young people who have come into contact with suicide experiences, as they live in areas where suicide plagues the life of the community and school, to elaborate on the meaning of this reality without suffering negative consequences, indeed developing more existential skills.

## 9. Conclusions

The death education intervention conducted with middle school students did not significantly impact all the variables investigated. However, we can say that it had an impact on enhancing life by promoting a positive view of the future, and it produced benefits on a personal level. Specifically, no deterioration was noted among participants, but their positive characteristics were stabilised, and their psychological dimensions saw an improvement. In particular, as hypothesised, decreased levels of alexithymia suggest an improvement in the ability to understand and communicate one’s emotions verbally and the preservation of psychological well-being and life satisfaction. Thanks to the use of artistic techniques, such as psychodrama, and the viewing of dedicated cartoons and photovoice, students improved their communication with their peers.

Despite these encouraging findings, some critical points need to be addressed in the future. In particular, no follow-up has been planned to assess whether the positive characteristics found in the experimental group remain constant or whether they are subject to further change. Moreover, it would be desirable to increase the number of participants in the intervention, involve a greater number of schools from different geographic areas and select the participants at random rather than, as in this case, because of needs identified by local political administrations.

### Limits of the Study

Despite these positive results, it is important to consider that the Cronbach’s alpha values for the EOT of the AQC are low, which could be due to the age of the participants and which requires us to read the results carefully. A further limitation is that the groups were not randomised due to the needs of the schools involved, which required that educational and training requirements be respected first and foremost. In fact, administrators and teachers gave permission to proceed with the project only on the condition that the interventions were conducted with classes that demonstrated particular needs and using as control groups only classes willing to undergo measurement as such.

## Figures and Tables

**Table 1:** Descriptive statistics of all the study variables by time for each group, with ANOVA results.

Measures	Experimental Group				Control Group				ANOVA Results ^a^		
	Pre-Test		Post-Test		Pre-Test		Post-Test				
	M	DS	M	DS	M	DS	M	DS	Group	Time	Time x Group
Alexithymia Questionnaire for Children (ACQ)											
Difficulty in Identifying Feelings (DIF)	0.57	0.37	0.50	0.38	0.54	0.35	0.48	0.39	0.26	5.46 *	0.00
Difficulty in Describing One’s Feelings (DDF)	0.87	0.43	0.76	0.44	0.69	0.42	0.73	0.48	2.47	0.92	5.22 *
Externally Oriented Thinking (EOT)	0.92	0.31	0.85	0.30	0.80	0.30	0.83	0.28	2.86	1.09	4.69 *
Hopelessness Scale for Children (HSC)											
HSC Total Score	4.61	2.84	4.28	2.63	4.28	2.69	4.38	2.90	0.09	0.36	1.27
Resilience Scale for Adolescents (READ)											
Family Cohesion (FC)	4.02	0.74	3.99	0.75	4.25	0.65	4.12	0.72	2.86	2.88	0.94
Social Competence (SC)	3.77	0.74	3.70	0.60	3.99	0.60	3.81	0.79	2.61	7.29 **	1.06
Personal Competence (PC)	3.74	0.72	3.74	0.71	3.84	0.72	3.81	0.69	0.64	0.11	0.21
Social Resources (SR)	4.22	0.68	4.03	0.78	4.48	0.53	4.27	0.62	6.32 *	18.93 ***	0.10
Goal Orientation (GO)	4.14	0.72	4.05	0.73	4.15	0.65	4.21	0.62	0.78	0.07	1.77
Stirling Children’s Well-being Scale (SCWBS)											
SCWBS Total Score	45.58	7.99	45.80	7.63	46.53	7.97	46.10	8.19	0.27	0.04	0.45

^a^ Degrees of freedom are 1 and 148 for all F-test * *p* < 0.05; ** *p* < 0.01; *** *p* < 0.001.

**Table 2:** Correlations between all measures at the pre-test.

Measures	1	2	3	4	5	6	7	8	9
1. Difficulty in Identifying Feelings (DIF)									
2. Difficulty in Describing One’s Feelings (DDF)	0.49 ***								
3. Externally Oriented Thinking (EOT)	0.03	0.20 *							
4. HSC Total Score	0.38 ***	0.33 ***	0.25 **						
5. Family Cohesion (FC)	−0.09	−0.18 *	−0.29 ***	−0.32 ***					
6. Social Competence (SC)	−0.15	−0.18 *	−0.28 ***	−0.32 ***	0.35 ***				
7. Personal Competence (PC)	−0.22 **	−0.22 **	−0.36 ***	−0.53 ***	0.54 ***	0.66 ***			
8. Social Resources (SR)	−0.06	−0.17 *	−0.24 **	−0.28 ***	0.67 ***	0.52 ***	0.54 ***		
9. Goal Orientation (GO)	−0.27 **	−0.23 **	−0.28 **	−0.54 ***	0.44 ***	0.54 ***	0.72 ***	0.40 ***	
10. SCWBS Total Score	−0.23 **	−0.24 **	−0.25 **	−0.47 ***	0.66 ***	0.52 ***	0.76 ***	0.56 ***	0.60 ***

* *p* < 0.05; ** *p* < 0.01; *** *p* < 0.001.

**Table 3:** Moderation effect of death education intervention on the impact of global alexithymia change on student’s well-being and resilience at the post-test.

Predictor	Well-Being at the Post-Test			Resilience at the Post-Test														
				Family Cohesion			Social Competence			Personal Competence			Social Resources			Goal Orientation		
	B	SE	t	B	SE	t	B	SE	t	B	SE	t	B	SE	t	B	SE	t
Measure at the pre-test ^1^	0.70	0.06	12.69 ***	0.67	0.07	9.69 ***	0.72	0.06	11.66 ***	0.72	0.06	12.22 ***	0.81	0.08	10.25 ***	0.57	0.07	7.93 ***
Global alexithymia change	−0.36	1.01	−0.35	−0.02	0.09	−0.26	0.14	0.09	1.54	−0.02	0.24	−0.20	−0.04	0.10	−0.44	0.01	0.10	0.15
Death education intervention (1 = Yes; 0 = No)	−0.41	0.90	−0.46	0.03	0.09	0.39	0.01	0.08	0.07	−0.04	0.08	−0.51	−0.08	0.09	−0.87	−0.19	0.09	−2.14 *
Interaction (global alexithymia change by death education intervention)	3.72	1.38	2.70 **	0.16	0.13	1.23	−0.08	0.12	−0.68	0.28	0.12	2.37*	0.19	0.14	1.36	0.12	0.14	0.88

^1^ The corresponding variable at the pre-test for each dependent variable is included. * *p* < 0.05; ** *p* < 0.01; *** *p* < 0.001.
